# Human preterm colostrum stimulates outgrowth in neurogenic tissue

**DOI:** 10.1038/s41390-023-02721-z

**Published:** 2023-07-11

**Authors:** Julian Kaps, Veronica S. Georgieva, Laura Oberholz, Angela Kribs, Bent Brachvogel, Titus Keller

**Affiliations:** 1 Department of Pediatrics and Adolescent Medicine, Medical Faculty of the University of Cologne, Experimental Neonatology, Cologne, Germany; 2 Center for Biochemistry, Medical Faculty of the University of Cologne, Cologne, Germany; 3 Department of Pediatrics and Adolescent Medicine, Center for Neonatology and Pediatric Intensive Care Medicine, Medical Faculty of the University of Cologne, Cologne, Germany

## Abstract

**Background:**

The olfactory bulb has a key role for nasal delivery of drugs to the brain by its access from the nasal mucosa and its connection to the subventricular zone. The aim of this study was to investigate the neuromodulatory capacity of human milk of premature infants on the olfactory bulb.

**Methods:**

Olfactory bulbs from P1 mice were embedded in a collagen I gel and incubated with DMEM supplemented with the aqueous phase of human colostrum (Col) of five mothers after very preterm birth, mature milk (Mat) of the same mothers or without supplement (Ctrl). After 7 days, the neurite outgrowth was quantified. Proteome analysis of the milk samples was performed using unlabeled mass spectrometry.

**Results:**

Outgrowth increased significantly in bulbs exposed to Col but not when exposed to Mat. Mass spectrometry revealed profound differences in the proteome of Col versus Mat. Among 21 upregulated proteins in Col were proteins involved in neurite outgrowth, axon guidance, neuromodulation and longevity.

**Conclusions:**

A high bioactivity of human preterm colostrum on murine neonatal neurogenic tissue is demonstrated to be associated with a proteome profoundly differing from mature milk.

**Impact:**

- The hypothesis has been raised that neonatal brain damage in a preterm infant could potentially be ameliorated by intranasal application of maternal breast milk.
- In an in-vitro model using neonatal murine olfactory bulb explants a significant stimulatory effect by human preterm colostrum is observed.
- Proteomics reveals upregulated neuroactive proteins in human colostrum compared to mature milk.
- A confirmation of this exploratory study would indicate that preterm colostrum stimulates neurogenic tissue.
- Early intranasal colostrum application might attenuate perinatal loss of neurogenic tissue thereby contributing to reducing complications such as cerebral palsy.

## Introduction

Breast milk feeding of preterm infants is associated with less severe adverse neurodevelopmental outcome despite an increased risk for suboptimal weight gain, described as the apparent breastfeeding paradox.^1^ It is known that human milk contains neurotrophins and other growth factors such as EGF.^2^ Neurotrophins are known to modulate neurogenesis and their intranasal delivery mediates neuroprotective and regenerative effects in animal models of neonatal brain injury.^3^ Specifically, in cases of severe intraventricular hemorrhage (IVH) in preterm infants new strategies are needed to protect the vulnerable brain during the high risk phase for loss of neurogenic tissue.^4^ Considering the complex interactions during breastfeeding such as, oxytocine modulated maternal infant bonding promoting offspring cognitive development, it is difficult to evaluate the isolated effect of the mothers’ own milk (MOM) on neurocognitive development in a clinical study.

Therefore, an in-vitro model was needed to safely study the isolated effects of MOM on the neonatal brain and elucidate the role of its composition. We present an in vitro model using murine neonatal olfactory bulb (OB) explants to examine effects of human milk on neonatal neurogenic tissue.

## Methods

### Milk preparation

MOM samples were collected from five infants, born with gestational age 25 to 28 weeks at the neonatal center of the University Hospital of Cologne with informed consent and approval by the local Ethical board (EK 15–368).^5^ Colostrum samples (2 ml) were collected before the onset of lactogenesis II confirmed by color, volume and electrolyte concentrations (90–105 h postnatally, sodium 21–29 mmol/l) and the mature milk samples (10 ml) collected 14 days postnatally. Within 4 h each sample was centrifuged at 16.000 g for 5 min to isolate the aqueous phase, which then was stored at −80 °C. After thawing for the in vitro experiments, the colostrum samples of donor 1–3 (pCol) and the mature milk samples (pMat) were pooled. For confirmatory experiments, single colostrum samples (donors 4 and 5) were used.

### Mice

C57BL/6 N mice were used for the animal studies. All experiments were performed in accordance with the ethics guidelines of the German animal protection law. Institutional review board - Landesamt für Natur, Umwelt und Verbraucherschutz Nordrhein-Westfalen.

### In vitro model for neurite outgrowth assessment

For neurite outgrowth assessment, collagen I gels were prepared freshly by adding rat tail collagen I (0.76 mg/mL, First Link UK Ltd., UK), ascorbic acid (0.61 mg/mL, Sigma-Aldrich, Germany) and NaOH (31 mM, VWR) to DMEM (Gibco, Thermo Fisher Scientific). Olfactory bulbs were explanted from one litter of P1 C57BL/6 N mice and each bulb was placed in 400 µL collagen gel in 24-well plates. After polymerization of the gel at 37 °C and 5% CO_2_, 400 µL DMEM either without any supplements or with 10% (v/v) pCol or pMat milk were carefully added. The bulbs were incubated for 7 days at 37 °C and 5 % CO_2_ and analyzed with a stereo microscope (Nikon SMZ 1500, Japan). The ratio between neurite area and bulb area was determined using ImageJ software.

### Mass spectrometry and Gene Ontology enrichment analysis

Approximately 50 µg of protein per sample were reduced, alkylated and digested in Lys-C and trypsin. The samples (*n* = 3 biological replicates) were analyzed on a liquid-chromatography mass-spectrometer coupled to a nano-flow liquid chromatograph (Thermo Scientific) following standard protocols. Peptides were identified by searching expected protein sequences in the UniProt Human database (71,931 entries, downloaded from Uniprot 16.6.2017) using MaxQuant software (version 1.5.3.8, https://www.maxquant.org/).

LFQ intensities were loaded into Perseus software (version 1.6.1.1, https://www.maxquant.org/perseus). A two sample Student’s *t*-test was performed using permutation-based FDR estimation. Proteins with a *q*-value below 0.05 (26 in total) were considered significantly different between the two groups. Data were imported in InstantClue (version 0.5.34) for visualization by principal component analysis and volcano plot. Gene Ontology term analysis was performed according to the PANTHER Classification System (https://geneontology.org).

### Statistical analysis

Significance between multiple groups was determined by one-way ANOVA analysis and subsequent Tukey’s multiple comparisons test. For comparison of two groups, the unpaired *T*-Test, two-tailed, was used. *P*-values of *p* < 0.05(*) were considered as significant. Mean values and standard deviations are shown in the graphs (GraphPad Prism, version 9.00).

## Results

### Neurotrophic potential of human breast milk

**Fig. 1:**
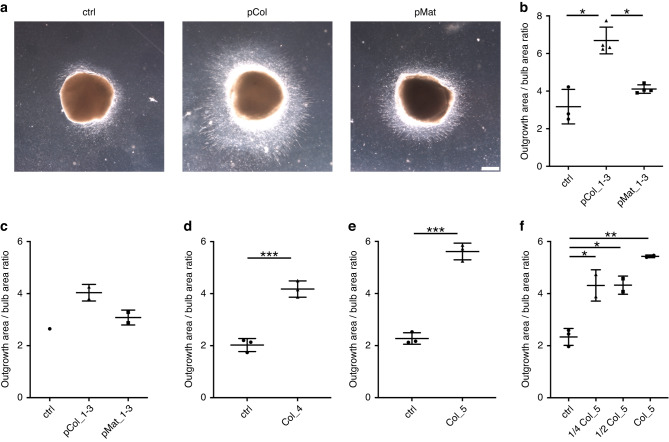
Stimulation of outgrowth by human milk. **a** Murine olfactory bulb explants were incubated without milk (ctrl), with colostrum (Col) and mature milk (Mat), and the outgrowth was analyzed by light microscopy after 7 days. Representative images are shown. Scale bar: 500 µm. **b**–**f**) Quantification of the neurite area to bulb area ratio is shown. Each graph represents one experiment with one litter of mice. **b**, **c** Pooled samples are from donor 1, 2 and 3. **d** Colostrum of donor 4. **e** Colostrum of donor 5 and (**f**) dilution experiment with colostrum of donor 5.

Olfactory bulb (OB) explants were cultured for 7 days in the presence of pooled colostrum of three donors (pCol), pooled mature milk (pMat) or in medium without supplements (ctrl) (Fig. 1a). Outgrowth quantified by the ratio of neurite area to bulb area revealed a 2.1-fold increase in outgrowth in pCol-treated bulbs compared to control (*p* = 0.021), the outgrowth of pMat-treated cultures was 1.3-fold (ns) (Fig. 1b). This increase in outgrowth in the presence of colostrum was confirmed in 4 additional litters and with colostrum of two further donors (Fig. 1c–f). Moreover, we demonstrated that the stimulatory effect on outgrowth was detectable in presence of lower concentrations of colostrum (Fig. 1f). The neurostimulatory effect was also shown in a live cell imaging experiment using the DNA-dye Hoechst 33,342. Here radial cell migration within the outgrowth area was observed associated with increased cell proliferation (Video suppl.). The results demonstrate that preterm human colostrum stimulates outgrowth out of the olfactory bulb significantly, including radial cell migration and proliferation.

### Mass spectrometry analysis of human breast milk components

**Fig. 2:**
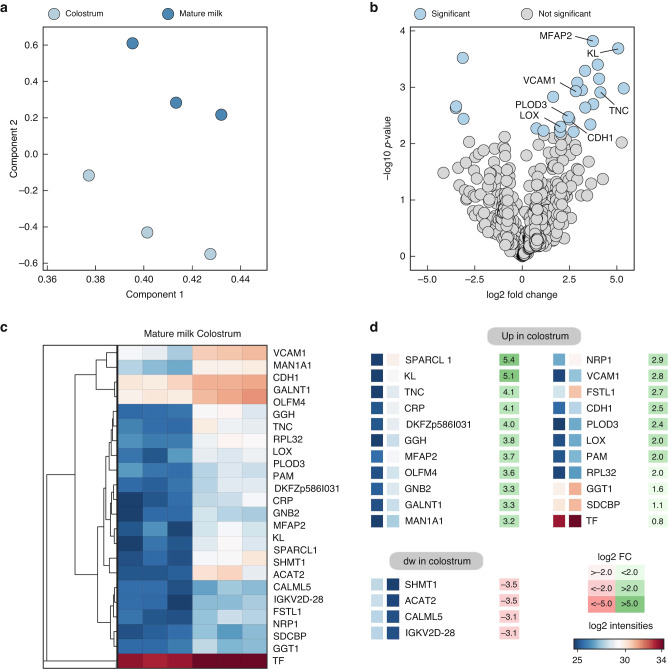
Proteome analysis of human breast milk samples. Colostrum and mature milk samples were analyzed by mass spectrometry (*n* = 3 per biological replicate). **a** Principal component analysis of the proteome dataset. Each circle represents an individual breast milk sample. **b** Volcano plot. Significant changes within the dataset are indicated (blue). Proteins with the highest fold change are annotated. **c** Heatmap of significantly regulated milk proteins. **d** List of up- and downregulated milk proteins sorted according to fold change.

Principal component analysis using mass spectrometry revealed substantial differences between the colostrum versus mature milk used in our experiments (Fig. 2a). In total, 516 proteins were identified and among those, 26 differed significantly (Fig. 2b). 21 proteins were enriched in colostrum (Fig. 2d). The most highly upregulated proteins were hevin (5-fold), klotho (5-fold) and tenascin C (4-fold). A higher abundance of proteins involved in neuron axon guidance (neuropilin 1), in neuromodulation (syntenin 1), and cell adhesion (cadherin 1 and vascular cell adhesion molecule 1).

### Highly upregulated neuroactive proteins in Colostrum

According to GeneOntology term analysis, most of these proteins are secreted extracellular space components. Five of these colostrum proteins are involved in synapse organization (neuropilin-1, cadherin-1, tenascin C, hevin and syntenin-1). Six are involved in extracellular matrix organization (Procollagen-lysine,2-oxoglutarate 5-dioxygenase 3, Microfibrillar associated protein 2, Cadherin-1, Vascular cell adhesion protein 1, Tenascin C, Lysyl oxidase).

## Discussion

Our results show that human colostrum has a strong stimulatory effect on outgrowth of neurogenic tissue using the murine neonatal olfactory bulb explant system. A molecular signature of neuroactive proteins was identified in colostrum compared to mature milk by proteome analysis.

The effect of colostrum on the olfactory bulb tissue is remarkable since the OB is a neurogenic niche receiving neural stem cells from the subventricular zone (SVZ) via the rostral migratory stream (RMS). Scranton et al. demonstrated the relevance of the RMS for the intranasal route into the brain by proving the connection between the OB and the SVZ in both directions.^6^ Intranasal neurotrophins induce neurogenesis in the murine SVZ.^7^ Clinical trials on intranasal insulin have demonstrated efficiency and safety for intranasal delivery of growth factors into the brain in human adults.^8^ Sanai et al. demonstrated the existence of the RMS in humans with strongest prominence in infancy.^9^

Here, the aqueous phase of human preterm colostrum induced the increased OB outgrowth significantly. The higher bioactivity of colostrum compared to mature milk is well known and mostly attributed to higher levels of immunoglobulins and growth factors. Neuroprotective effects by bovine colostrum in a rat model of intracerebral hemorrhage was reported by Kim et al. including significant suppression of apoptosis.^10^ Our results suggest that colostrum after preterm birth might mediate a similar effect.

The milk proteomics revealed highly upregulated neuroactive proteins in colostrum. Among these, for the longevity hormone klotho increased plasma-levels are associated with improved cognitive performance in mice and humans.^11^ Hevin is involved in the regulation of Insulin-like Growth Factor transport and uptake.^12^ Neuropilin-1 plays versatile roles in the regulation of neurogenesis, angiogenesis, axon guidance, cell survival and remyelination and is important in tissue remodeling following brain injury.^13^ Tenascin C is highly expressed in the extracellular matrix during development and in the adult central nervous system (CNS) in regions of active neurogenesis.^14^ Soluble Vascular Adhesion Molecule-1 modulates the Blood Brain Barrier permeability. Follistatin-like 1 is involved in CNS development and Cadherin-1 in synapse development and plasticity. Sequential testing of the candidate proteins in the OB model might identify the main factor for the neuromodulatory effect, although the composition may be responsible.

Our observation is of high clinical relevance, since the increased bioactivity of MOM coincides with a high vulnerability of the neurogenic niche during the first postnatal days with the peak incidence for germinal matrix /IVH in very preterm infants. In a clinical study, fresh MOM which also contains living cells was applied intranasally to preterm infants after diagnosis of severe IVH and a trend for less severe residual brain defects at discharge was reported.^15^ We hypothesize that an earlier intranasal exposure to fresh colostrum might increase beneficial effects on the brain.

In conclusion, our results suggest a high bioactivity of human preterm colostrum on murine neonatal neurogenic tissue associated with a proteome profoundly differing from mature milk. A better knowledge on the neuromodulatory effects of human colostrum for the neurogenic niche of preterm infants will help neonatologists to improve existing strategies and to develop new approaches to ameliorate neonatal brain injury.

## Data Availability

The data that support the findings of this study are available from the corresponding author, T.K., upon reasonable request.
